# The evaluation of pulmonary function and blood gas analysis in patients submitted to laparoscopic versus open nephrectomy

**DOI:** 10.1590/S1677-5538.IBJU.2015.0040

**Published:** 2015

**Authors:** Ayfer Koc, Gozde Inan, Fusun Bozkirli, Demet Coskun, Lutfi Tunc

**Affiliations:** 1 Department of Anesthesiology and Reanimation, Gazi University Faculty of Medicine, Ankara, Turkey; 2 Department of Urology, Gazi University Faculty of Medicine, Ankara, Turkey

**Keywords:** Laparoscopy, Nephrectomy, Anesthesia

## Abstract

**Background:**

The aim of this study was to assess the early postoperative pulmonary function and arterial blood gases in patients who have undergone open versus laparoscopic nephrectomy.

**Materials and Methods:**

Forty patients were randomly assigned to undergo laparoscopic (LN, n=20) or open nephrectomy (ON, n=20). Pulmonary function tests including forced expiratory volume in 1 second (FEV_1_), forced vital capacity (FVC), forced expiratory volume at 25% (FEF_25_), forced expiratory volume at 50% (FEF_50_), forced expiratory volume at 25% to 75% (FEF_25–75_), forced expiratory volume in 1 second (FIV1) and peak expiratory flow (PEF) were performed one day before the operation and on the postoperative day 1. The arterial blood gas analysis (pH, pCO_2_, pO_2_, SaO_2_) was made at breathing room preoperatively, in the recovery phase and on postoperative day 1.

**Results:**

All spirometric variables decreased after both open and laparoscopic nephrectomy on postoperative day 1. FEV_1_, FVC, FEF_25_ and FEF_25–75_ values decreased on postoperative day 1 (39.7%, 37.4%, 27.7%, 51.8% respectively) in the open surgery group and they were significantly lower in laparoscopic group (29.9%, 32.5%, 23.2%, 44.5% respectively). There were no significant differences in FEF_50_, PEF and FIV_1_ between the groups. The SaO_2_ and pO_2_ values also decreased in both groups. During early recovery, pH decreased while pCO_2_ increased significantly but they returned to preoperative values on postoperative day 1 in both groups.

**Conclusion:**

Laparoscopic nephrectomy is better than open nephrectomy considering pulmonary functions.

## INTRODUCTION

Pulmonary complications like hypoxemia, atelectasis and pneumonia are the main causes of morbidity after abdominal surgery (1, 2). Upper abdominal surgeries are more associated with pulmonary impairment, which seems to be related with diaphragmatic dysfunction (3, 4). Laparoscopic surgery has been the method of choice over open techniques with numerous advantages such as reduced perioperative bleeding, less post-operative pain, less injury to the abdominal wall muscles and may have less effect on pulmonary functions (5, 6).

Like other laparoscopic surgeries, laparoscopic nephrectomy offers multiple advantages over open nephrectomy including reduced postoperative morbidity and pain, rapid recovery, shorter hospital stays and faster discharge (7, 8). Moreover, significant reduction in the probability of adverse events and improvement of perioperative safety outcomes were observed in laparoscopic compared open nephrectomy with two observational patient safety studies, respectively (8, 9).

In literature, numerous studies compare pulmonary functions among various open and laparoscopic upper abdominal surgery techniques involving cholecystectomy (4, 5, 10), gastric bypass (6), colectomy (11), and esophagogastric surgery (3) but we were not able to find any investigations related to nephrectomy. In the present study under general anesthesia with isoflurane and remifentanil and providing a good postoperative analgesia, we examined and compared impairment of pulmonary functions after laparoscopic and open nephrectomy using spirometric tests and arterial blood gas analysis. The aim of this study was to assess the early postoperative pulmonary functions and to show any superiority for laparoscopic over open nephrectomy.

## MATERIALS AND METHODS

The Ministry of Health, Turkey, General Directorate of Pharmaceuticals and Pharmacy ethics board approved this study. The study included American Society of Anesthesiology (ASA) I-II and III risk groups of forty 27–65 years old patients with renal cell carcinoma with no history of lung disease and with normal lung function tests. After the patients gave an informed consent, they were randomized in one of two groups using a computer generated table of random numbers, open (ON, n=20) or laparoscopic (LN, n=20) nephrectomy. To evaluate pulmonary function, forced expiratory volume in 1 second (FEV_1_), forced vital capacity (FVC), forced expiratory volume at 25% (FEF25), forced expiratory volume at 50% (FEF5_0_), forced expiratory volume at 25% to 75% (FEF_25–75_), forced expiratory volume in 1 second (FIV_1_) and peak expiratory flow (PEF) as determined by spirometry and arterial blood gas analysis (pH, PaCO_2_, PaO_2_, SaO_2_) were used. Pulmonary function tests of the patients were performed in standing posture, at rest before and on first day after the operation with a mobile hand spirometer (Spirobank G, MIR, Via del Maggiolino 12500155 ROME). Arterial blood gases were analyzed during rest and 1 hour and 24 hours after the operation. Standard general anesthesia consisting of inhalation anesthesia with isoflurane and adding remifentanil infusion was administered to all patients.

Analgesic protocol was the same for all patients and early postoperative analgesia was provided using intravenous 20mg tenoxicam and 0.25mg kg^−1^meperidine. Within the first 24 hours, postoperative pain was assessed using a visual analogue scale (VAS, 0=no pain, 10=maximum pain) and whenever score was >3 meperidine (1mg kg^−1^ intramuscular) was administered. Pulmonary function tests were not performed unless VAS score was under 3.

Maximum intra-abdominal pressure was limited to 14mmHg with CO_2_ insufflation in the LN group. Abdominal incision was performed for ON operations. The same anesthesia and surgeon team performed all procedures.

Transperitoneal laparoscopic nephrectomy was performed with descending technique (also called Tunc technique). The patients were maintained in the lateral decubitus position where they were in a semi-oblique position with an angle of 70° with the bed. For nephrectomy, only three trocars were used. The first trocar was placed 3–5cm superior to the umbilicus and 3–4cm lateral to midline. The second trocar was 10–15cm lateral to the umbilicus. The third trocar was 3–5cm superior to the first trocar and 1cm lateral. These locations can change according to the operation side. Open radical nephrectomy was performed with a flank incision. At either method colon was moved away from the kidney, the renal hilum was identified and dissected. First renal artery and then renal vein were clamped and cut. Proximal 1/3 ureter was dissected, clamped and cut. Nephrectomy specimen contained the adrenal gland in case of upper pole tumors. In the laparoscopic nephrectomy group, the specimen was removed with an endobag. The duration of surgery was defined as the time interval from skin incision in the open nephrectomy group or first trocar placement in the laparoscopic group to closure in minutes.

Data analysis was performed using SPSS 17.0 statistical program and summarized as mean±SD, n or %. The Kolmogorov-Smirnov test was used to show a normal distribution of the data. Demographic data, duration of operation and anesthesia, VAS and pulmonary function tests were compared using Student t test between groups. Intra-group comparison of continuous data was evaluated with repeated measures of variance test and when a significant difference was present, a Bonferroni correction was made. Paired sample t test was used to compare intra-group differences in the results of pulmonary function tests and VAS. Gender and ASA were compared using chi-squared or Fisher's exact chi-squared tests. P values less than 0.05 were considered statistically significant.

## RESULTS

There were no significant differences between the groups regarding age, sex, weight, height and ASA classification (Table-1). Duration of surgery varied from 147.21min to 98.0min (p<0.05) for ON and LN, respectively, with duration of anesthesia also being shorter for the LN group.

**Table 1:** Patient characteristics and duration features (mean±standard deviation (min-max).

	Laparoscopic Nephrectomy (n=20)	Open Nephrectomy (n=20)	p
**ASA (I/II/III)**	3/12/5	5/13/2	0.401
**Gender (M/F)**	11/9	11/9	1.000
**Age (year)**	55.15±9.73 (29–65)	51.10±13.65 (27–65)	0.287
**Weight (kg)**	74.00±10.98 (55–96)	69.60±11.55 (52–96)	0.224
**Height (cm)**	165.80±9.50 (150–186)	162.63±9.45 (145–178)	0.303
**Duration of operation (min)**	98.00±24.98 (60–133)	147.21±36.35* (88–195)	<0.0001
**Duration of anesthesia (min)**	111.90±25.27 (70–150)	158.74±36.82* (100–207)	<0.0001

**ASA =** American Society of Anesthesiologists

* p<0.05 in comparison with laparoscopic group

Preoperative and postoperative first day mean pulmonary function test results are listed in Table-2. In both groups, a decrease in all spirometric variables was detected on the postoperative day 1. A decrease of 39.7%, 37.4%, 27.7%, 51.8% in Group ON and 29.9%, 32.5%, 23.2%, 44.5% in Group LN was detected for FEV_1_, FVC, FEF_25_ and FEF_25–75_, respectively, when compared to preoperative values. The difference was significantly lower in Group LN (p<0.05). PEF and FIV_1_ showed the same tendency in both groups without a significant difference between the two groups.

**Table 2:** Spirometric data as percentages of individual predictive values comparing laparoscopic and open nep hrectomy [mean (standard deviation)].

		Laparoscopic Nephrectomy (n=20)	Open Nephrectomy (n=20)	p
FEV_1_ (%)	**Preoperative**	87.1 (27.7)	79.5 (27.3)	0.362
	**Postoperative 1st day**	57.2 (27.4)†	39.8 (17.7)* †	0.025
FVC (%)	**Preoperative**	91.2 (23.3)	80.2 (28.9)	0.192
	**Postoperative 1st day**	58.7 (27.5)†	42.8 (19.0)* †	0.043
FEF_25_ (%)	**Preoperative**	65.4 (25.8)	54.0 (26.7)	0.177
	**Postoperative 1st day**	42.2 (22.0)†	26.3 (14.9)* †	0.012
FEF_50_ (%)	**Preoperative**	69.5 (23.5)	57.1 (27.1)	0.135
	**Postoperative 1st day**	42.3 (23.1)†	34.0 (27.7)†	0.324
FEF_25–50_ (%)	**Preoperative**	92.8 (36.0)	84.1 (35.7)	0.452
	**Postoperative 1st day**	48.3 (24.1)†	32.3 (19.9)* †	0.027
PEF (%)	**Preoperative**	70.2 (24.6)	57.2 (26.2)	0.118
	**Postoperative 1st day**	51.0 (24.5)†	38.2 (23.9)†	0.112
FIV_1_ (%)	**Preoperative**	94.6 (27.8)	82.0 (36.2)	0.233
	**Postoperative 1st day**	43.8 (15.8)†	52.3 (24.0)†	0.236

**FEV_1_ =** forced expiratory volume in 1 second; **FVC =** forced vital capacity; **FEF_25_,** = forced expiratory volume at 25%; **FEF_50_ =** forced expiratory volume at 50%; **FEF_25-75_ =** forced expiratory volume at 25% to 75%; **FIV_1_ =** forced expiratory volume in 1 second; **PEF =** peak expiratory flow

* = p<0.05 in comparison with laparoscopic group

† = p<0.05 in comparison with preoperative values

Blood gas data are presented in Table-3. There were no statistically significant differences between the two groups for all parameters of blood gas analysis. There was a significant difference (p<0.05) in pH, which decreased, and pCO_2_, which decreased, in the first hour following the operation; however, they both returned to the preoperative levels on postoperative first day. A significant increased (p<0.05) in SaO_2_ and pO_2_ values was documented on postoperative day 1 in both groups.

**Table 3:** Values of arterial blood gas analysis comparing laparoscopic and open nephrectomy [mean (standard deviation)].

		Laparoscopic Nephrectomy (n=20)	Open Nephrectomy (n=20)	p
**PaCO_2_**	**Preoperative**	32.69 (4.50)	32.73 (4.21)	0.978
	**Postoperative 1st hour**	36.68 (4.08)†	35.30 (5.25)†	0.357
	**Postoperative 1st day**	32.82 (3.93)	32.02 (3.72)	0.523
**PaO_2_**	**Preoperative**	73.89 (8.18)	77.15 (7.04)	0.185
	**Postoperative 1st hour**	85.02 (34.41)	94.65 (41.61)	0.430
	**Postoperative 1st day**	68.25 (8.90)†	69.70 (8.84)†	0.617
**SaO2**	**Preoperative**	95.35 (1.53)	95.59 (1.95)	0.668
	**Postoperative 1st hour**	95.43 (2.60)	94.98 (2.90)	0.608
	**Postoperative 1st day**	94.17 (1.91)†	93.27 (3.59)†	0.343
**pH**	**Preoperative**	7.42 (0.03)	7.43 (0.02)	0.290
	**Postoperative 1st hour**	7.36 (0.04)†	7.36 (0.06)†	0.776
	**Postoperative 1st day**	7.42 (0.03)	7.42 (0.03)	0.622

† = p<0.05 in comparison with preoperative values

The VAS pain scores were similar in both groups half an hour, an hour and 24 hours after the operation (Table-4).

**Table 4:** VAS scores [mean±standard deviation (min-max)].

	Laparoscopic Nephrectomy (n=20)	Open Nephrectomy (n=20)	p
**Postoperative 30th minute**	3.80±1.28 (0–5)	3.80±0.70 (3–5)	1.000
**Postoperative 1st hour**	2.85±1.22 (0–5)†	2.95±0.69 (2–4)†	0.752
**Postoperative 1st day**	1.25±0.91 (0–2)†	1.30±0.80 (0–2)†	0.855

**VAS =** Visual analogue scale

† = p<0.05 in comparison with preoperative values

## DISCUSSION

Upper abdominal surgeries have been known to have more pronounced effect on pulmonary functions than lower abdominal surgeries, mostly attributed to postoperative pain and diaphragm dysfunction (4, 12). Laparoscopic techniques seem to cause less damage to abdominal wall and less postoperative pain, this is why less pulmonary complications are considered (5, 6). Comparison of pulmonary functions in laparoscopic versus open abdominal surgeries have been well studied (3–6, 10–15). So, we hypothesized that improved postoperative pulmonary function described after various laparoscopic surgeries could be expected similarly to benefit patients undergoing nephrectomy. Our findings for laparoscopic and open nephrectomy are in line with other data. We similarly showed that considerable changes in pulmonary function occurred after both laparoscopic and open nephrectomy but these were less pronounced after LN than ON.

In patients undergoing abdominal surgery, besides maintaining basic principles of general anesthesia, there should be a good relaxation of abdominal wall tonus, and suppression of sympathetic and hemodynamic reflexes, and perfect postoperative analgesia should be provided (16). General anesthesia itself has an important role in impairment of pulmonary functions due to disturbance of gas exchange (17). This effect of general anesthesia lasts short and mostly return to baseline values within a day (5, 10). However, independent from the effects of general anesthesia, upper abdominal surgery has more pronounced and long-lasting effects on pulmonary functions that are characterized in restrictive patterns (15).

Moreover, as operation and anesthesia durations last longer, the decline in pulmonary function test parameters gets greater. Osman et al. (10) in their comparative study observed a rapid decrease in FEV_1_ if anesthesia lasted longer than 60 minutes and in that study, anesthesia lasted 102 minutes in open cholecystectomy and 57 minutes in laparoscopic cholecystectomy group. In the present study, mean duration of anesthesia was noted as 111.90 minutes for the laparoscopic group and 158.74 minutes for the open group, which are statistically significant. Even though the above mentioned authors suggested that laparoscopy has more advantages as it provides shorter duration of anesthesia, longer anesthesia duration of open nephrectomy group in our study may represent a limitation to the study.

In the present study comparing early pulmonary functions in laparoscopic versus open nephrectomy, in line with results of other upper abdominal surgery studies, considerable changes were noted after both LN and ON, although these were less pronounced after ON than those after LN. FEV_1_ and FVC were more suppressed in patients undergoing open nephrectomy (p<0.05). FEV_1_ and FVC decreased by 49.93% and 46.63%, respectively, in ON and 34.32% and 35.63%, respectively, in LN, when compared to their preoperative values (p<0.05). Similar changes occurred in FEF_25_ and FEF_25–75_. There were no significant differences concerning FEF_50_, PEF and FIV_1_. Crema et al. (3) compared pulmonary dysfunction in patients who underwent open and laparoscopic esophagogastric surgery and demonstrated a 25.2% and 27.6% reduction in open and a 13.7% and 15.7% reduction in laparoscopic group for FEV_1_ and FVC, respectively, on postoperative second day. Hasukic et al. (4) evaluated pulmonary functions in 60 patients who underwent either laparoscopic or open cholecystectomy and concluded that pulmonary functions were better preserved after laparoscopic resection. Shauer et al. (15) also demonstrated less impairment of postoperative pulmonary function after laparoscopic cholecystectomy than conventional open cholecystectomy. On postoperative first day, they found a decrease of 79% and 76% after open, and 49% and 44% after laparoscopic group in FVC and FEV_1_, respectively, when compared to their preoperative values. Nguyen et al. (6) also found less postoperative suppression of pulmonary function with laparoscopic gastric bypass than open gastric bypass. The diversity of reduction rate for spirometric parameters in literature can be explained by the variability of surgical methodologies, the site and size of incisions, and the severity of diaphragmatic dysfunctions.

There are two possible approaches for laparoscopic nephrectomy, transperitoneal or retroperitoneal. While both have individual benefits and disadvantages, anesthesia related main difference is bilateral intraperitoneal versus unilateral retroperitoneal CO_2_ insufflation. El-tohamy SA. et al. (18) demonstrated markedly higher PaCO_2_ at the same insufflation pressure and significantly greater peak airway pressure in transperitoneal compared to retroperitoneal CO_2_ insufflations maintaining the same minute ventilation. The authors concluded that ventilatory, hemodynamic and cerebral implications were more pronounced in transperitoneal nephrectomy than in retroperitoneal nephrectomy. Similarly, in an experimental study (19), to maintain normocapnea, significantly greater peak airway pressures were needed to administer an adequate tidal volume in intraperitoneally versus retroperitoneally CO_2_ insufflated animals. These findings are in accordance with a previous study that suggested a less pulmonary impairment with retroperitoneal technique (20). Even though retroperitoneal approach was associated with less pulmonary disturbance, the selection of the approach depended on the surgeon team in the present study and they preferred transperitoneal approach based on their experience. Anyway, both transperitoneal and retroperitoneal approaches were found excellent with no significant difference between the groups for postoperative complications in a comparative study and the approach to be selected was at the discretion of the surgeon.

In previous studies comparing laparoscopic open techniques, there is diversity in the results of blood gas analysis. We found no statistically significant differences between the two groups in all parameters of blood gas analysis. pH decreased at immediate recovery in both groups with an increase in pCO_2_ but all returned to preoperative levels on postoperative day 1. SaO_2_ and pO_2_ values reduced on postoperative day 1 when compared to preoperative values, however, this reduction was not significant between laparoscopic and open groups. Similarly, Hasukic et al. (4) found no significant changes in arterial pO_2_, pCO_2_ and pH at 24 h between laparoscopic and open cholecystectomy patients. On the contrary, Karayiannakis et al. (13) and Mimica et al. (14) reported better oxygenation in laparoscopic cholecystectomy than in open surgery. Despite this variability, laparoscopy is considered to deteriorate blood gas values to a lesser degree.

The incision used in open nephrectomy is painful and can be the reason for altered ventilation in those patients. In order to avoid bias, we used a strict protocol for the standardization of anesthesia management, care and analgesic use. Notably, the protocol of the present study mandated the maintenance of VAS<3 especially when the pulmonary function tests were performed. However, only early, first 24 hours and postoperative impairment of pulmonary functions were compared in the current study and it is difficult to identify the reasons of deterioration of pulmonary functions. Another limitation in our study is the lack of recording the amount of analgesics consumed and moreover the VAS pain scores were evaluated only at rest but scores might differ between the groups during coughing and mobilization.

## CONCLUSIONS

Early postoperative pulmonary functions are found to be less impaired in the laparoscopic nephrectomy group than in the open nephrectomy group, but the effects on arterial blood gases were similar in both groups and no clinically relevant differences were found. In the light of these findings, we concluded that laparoscopic nephrectomy would be superior to open nephrectomy considering pulmonary functions.
